# 

**Published:** 1879-10

**Authors:** 


### Table of Contents.

### ORIGINAL COMMUNICATIONS.

Trismus Nascentium. By. Prof. J. T. Banks, M.D., Atlanta, Ga..... 385
Reflex Nervous Troubles from Genito-Urinary Irritation, with Illustrative Cases. By R. W. Westmoreland, M.D., Atlanta, Ga...... 387

### REPORTS OF SOCIETIES.

Alleghany County Medical Society...... 392
Proceedings of the Cinc'nnati Academy of Medicine...... 394
Toledo Medical Association...... 399

### SELECTIONS

Gunshot Wound of Uterus; Bullet Traversing Six Months' Fœtus; Recovery of Patient in Four Weeks...... 409
Theories of Fever...... 412
Intravenous Injection of Ammonia...... 416
Antiseptic Surgery...... 420
History of Massage...... 426
Inflammation of the Pleura...... 435
Clinical Lecture on Facial Palsy...... 440

### EDITORIAL.

The Fever of Neighb)ring Counties...... 444

Medical Teaching...... 445

Errata...... 445

### BIBLIOGRAPHICAL.

The National Dispensatory...... 446

### EXCERPTA.

Mixture of Digitalis and Iron for Cardiac Weakness, with Dilation of Ventricles...... 446
How to Prevent Mammary Abscess...... 447
Regenera'ion of the Eye...... 448

